# Classroom air quality in a randomized crossover trial with portable HEPA air cleaners

**DOI:** 10.1038/s41370-025-00743-9

**Published:** 2025-01-11

**Authors:** Shayna C. Simona, Scott M. Bartell, Verónica M. Vieira

**Affiliations:** Department of Environmental and Occupational Health, Joe C. Wen School of Population & Public Health, University of California, Irvine, CA USA

**Keywords:** Air pollution, Particulate matter, Healthy buildings

## Abstract

**Background:**

Children living in communities with lower socioeconomic status and higher minority populations are often disproportionately exposed to particulate matter (PM) compared to children living in other communities.

**Objective:**

We assessed whether adding HEPA filter air cleaners to classrooms with existing HVAC systems reduces indoor air pollution exposure.

**Methods:**

From July 2022 to June 2023, using a block randomized crossover trial of 17 Los Angeles Unified School District elementary schools, classroom PM concentrations were monitored and compared for 99 classrooms with HEPA filter air cleaners and 87 classrooms with non-HEPA filter air cleaners.

**Results:**

In HEPA classrooms, average school-year PM_2.5_ was 39.9% lower (0.581 µg/m³; *p* < 0.001) and infiltration of outdoor PM_2.5_ into classrooms was 13.8–82.4% lower than non-HEPA classrooms, depending on the school.

**Impact:**

- Few studies have examined HEPA filtration in a classroom environment, and this is one of the first studies since the COVID-19 pandemic to assess PM exposure in the classroom. Using a well powered block randomized crossover trial, we showed that adding portable HEPA air cleaners to classrooms that already had HVAC systems with MERV 13 air filters resulted in lower measurable PM concentrations and less infiltration of outdoor PM_2.5_ compared to control classrooms with non-HEPA filters. This demonstrates that further improvements in classroom air quality, especially in environmentally burdened communities, can be achieved with additional filtration.

## Introduction

The California Air Resources Board’s (CARB) proposed Community Incentives 2019 guidelines include a funded program that incentivizes school districts to upgrade filtration in their classrooms by installing higher particle removal efficiency filters on existing heating, ventilation, and air conditioning (HVAC) systems or purchasing standalone high-efficiency particulate air (HEPA) purifiers. The aim of these measures was to reduce air pollution exposure in the classrooms, especially in Assembly Bill (AB) 617 communities in Los Angeles, California that experience greater environmental burdens. Children living in these communities, with lower socioeconomic status and larger minority populations, are disproportionately exposed and affected by particulate matter with diameters less than 2.5 micrometers (PM_2.5_) compared to children living in other communities [1, 2]. Los Angeles County is ranked within the top 25 counties with the most particle pollution in the nation [3].

Primary sources of particulate pollution in Los Angeles include motor vehicles, power plants, industrial facilities, oil refineries, port activity, and wild fires [4]. Indoor pollution in classrooms can originate from inside the building or infiltrate from outdoors through windows, doors, and ventilation systems. Coarse particles, such as PM_10_, include dust from construction sites and roads, and can irritate the eyes, nose, and throat. Smaller particles, like PM_1_ and PM_2.5_, can enter the blood or deep parts of the lungs [4]. Children are at higher risk for increased respiratory symptoms, such as irritation of the airways, coughing or difficulty breathing [5]. Furthermore, observational studies consistently show that poor classroom air quality is associated with reductions in cognitive performance and increases in short-term absence [6]. Since students inhale a larger volume of air corresponding to their body weights compared to adults, they are also at higher risk of health effects, including asthma exacerbation and worse respiratory symptoms, from air pollutants in school buildings [6].

The overall objective of this study was to assess if portable air purifiers using HEPA filters reduce classroom indoor air pollution exposure in environmentally disadvantaged communities using a randomized trial in Los Angeles Unified School District (LAUSD) elementary schools. Prior to the start of the study, LAUSD began using MERV13 filters in existing HVAC systems in response to the COVID-19 pandemic. This is one of the first studies since the COVID-19 pandemic to assess PM_2.5_ exposure in the classroom, where young students spend a substantial amount of time in one classroom.

## Material and methods

### Study design

A block randomized crossover trial was conducted to assess the benefits of portable HEPA air cleaners in 350 elementary school instructional classrooms at 17 elementary schools located in Carson, Torrance, Harbor City, and Lomita (within LAUSD in California). These schools were chosen because they are in South LA County, near the Port of Los Angeles, major highways, industries, and oil refineries, where air quality has historically been poor compared to other communities without these exposure sources. Classrooms within each school were randomly assigned to a treatment group (HEPA filters) or control group (non-HEPA filters) and air quality was measured in approximately half the classrooms (*n* = 186) due to costs. Classrooms were selected so that air quality was measured in each school building (permanent and portable), each school floor (if more than one story) and each end of the floor. Each classroom received the treatment for an entire school year, for two different school years (2022–2023 and 2023–2024). For the first school year, half the classrooms were randomly assigned to receive HEPA filters, and half the classrooms were assigned to receive non-HEPA filters. In the second school year of the study, the classrooms switched intervention groups (i.e., a cross-over randomized design). Each classroom also had an existing HVAC system maintained by LAUSD that used minimum efficiency reporting value (MERV) 13 filters since the COVID-19 pandemic; the portable air purifiers used in this study provided air filtration that was in addition to the existing HVAC filtration.

The first phase of the study was to assess whether there were any differences in air quality between the HEPA and non-HEPA classrooms. The classrooms were randomized per school and balanced across the two treatment groups, but schools with an odd number of classrooms received an additional HEPA classroom, which accounts for the difference between the number of control classrooms (*n* = 87) and intervention classrooms (*n* = 99) that were monitored. Intention to treat (ITT) analysis of the data was conducted to protect against potential noncompliance, protocol deviations, or anything that might have occurred after randomization. ITT analysis avoids overestimation of treatment effects (i.e., any bias from post-randomization factors is most likely towards the null) [7]. Compliance was not formally monitored. We present here results of PM monitoring in classrooms throughout the first year of the study.

PM concentrations were monitored using IQAir AirVisual Pro sensors, handheld devices that were placed inside classrooms. These sensors measure indoor air pollution from sources such as wildfire smoke and traffic pollution entering the building [8]. AirVisual Pro uses light-scattering to measure particles, converts the signal to a particle mass concentration for PM_1_, PM_2.5_, and PM_10_, and stores the data measured at 10-second intervals on the device memory [9]. The sensor showed very strong correlations with laboratory studies (R^2^ = 0.99) and overall showed 85–92% accuracy compared to reference instruments for concentrations <300 µg/m^3^ [10].

Blueair Classic 605 air cleaners were provided to the schools for this trial and used for both treatment groups (HEPA and non-HEPA). The air cleaner can filter room air at a rate of 4.8 times an hour (or about every 12.5 min) up to a 72 m² room [11]. These air cleaners have been used in other intervention studies to lower particulate concentrations [12]. We installed air cleaners with HEPA filters in classrooms that were randomized to treatment, and air cleaners with non-HEPA control filters in classrooms randomized to control. HEPA and non-HEPA filters were replaced every 6 months, during the schools’ summer and winter breaks, and the air cleaners were maintained as recommended by the manufacturer.

The air cleaners were placed in each classroom in mid-August 2022, just before the start of the school year. Baseline PM concentrations were measured in July prior to air cleaner installation. Teachers were instructed to always leave the air cleaners on the medium speed setting or higher and to not obstruct the air cleaner vents. They were also asked to leave the air monitors plugged in and accessible to researchers. The teachers and students were not informed of their randomized assignment, and the non-HEPA filters were designed to have a similar appearance to the manufacturer’s HEPA filters when installed in the air cleaners. Air cleaners for both treatment groups were sealed with tamper-evident tape after filter installation, to discourage teachers and students from opening the machines and revealing the filter type.

#### Statistical analysis

PM data were downloaded from classroom IQAir AirVisual Pro monitors during winter and summer breaks and reviewed for completeness. Monthly and school-year (September 2022 – May 2023) averages and standard deviations of the continuous PM concentrations were calculated for treatment and control groups and compared using the Welch Two Sample t-test. The monthly averages were only calculated for classrooms with data available for at least two weeks of the month. All daily measures were included in the average, including weekends and after-school hours. If the device was unplugged and did not collect data for more than two weeks for a month, their monthly average was excluded from the statistical analyses. Missingness was random across classrooms. We also fit a linear mixed effects (LME) model to data for the school-year months of the intervention. The model included PM_2.5_ as the outcome, with HEPA treatment as the main predictor of interest and was adjusted for baseline PM concentrations, with a random effect for school to account for any within-school correlation. We used R package ‘nlme’ version 3.1–164. We report the interclass correlation coefficient (ICC) for schools and the adjusted effect estimate for HEPA intervention on PM_2.5_.

Indoor classroom PM_2.5_ was also compared to outdoor PM_2.5_ collected at five schools with Clarity Node-S air monitors that were already in place prior to and independent of the intervention study. The sensors use light scattering to size and count particles and then convert them to a mass fraction and have performed well in field and lab evaluations [13]. Data from the outdoor air monitoring network are measured every 5–6 min and available online for download from the school district [14]. The PM_2.5_ data collected by the Clarity air monitor was averaged for school months September 2022 through May 2023. We estimated infiltration of outdoor air into each classroom by dividing the average indoor PM_2.5_ concentrations by the average outdoor PM_2.5_ concentration. We averaged the infiltration ratios and calculated confidence intervals by school for both treatment and control classrooms [12]. R 4.3.1 was used for data analysis. The Institutional Review Board at University of California, Irvine, and the Data Privacy, Analysis, & Reporting Branch and the Strategic Data and Evaluation Branch at LAUSD approved this study.

## Results

### Classroom air data

Our primary analysis examined the difference between indoor PM_2.5_ concentrations in classrooms with and without HEPA filters in portable air cleaners for schools with existing HVAC systems using MERV 13 filtration. The results of the LME model adjusting for baseline PM_2.5_ concentrations show that HEPA treatment classrooms were lower than control classroom by 0.390 µg/m³ on average with a standard error of 0.058 µg/m³. The ICC for school was low (0.15), supporting the approximate independence assumption of the *t*-tests, and indicating substantial variation in PM_2.5_ concentrations across classrooms within each school, compared to differences across schools. Many of the schools consist of a combination of main building classrooms and portable trailer classrooms with different proximity to roadways and other PM sources.

**Fig. 1:**
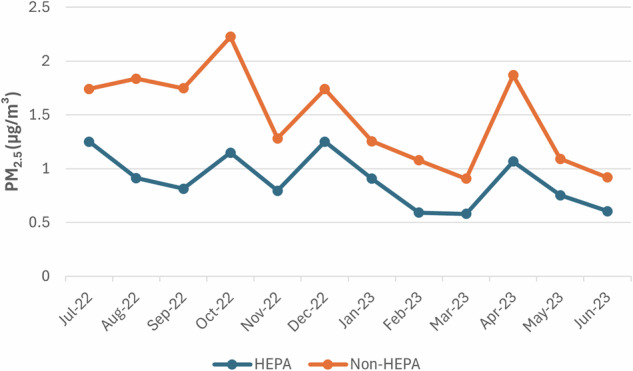
Temporal trends for indoor PM_2.5_. Monthly indoor PM_2.5_ (µg/m³) levels over the 2022–23 school year.

Table 1 shows the monthly and school year means and standard deviations (SD) for PM_2.5_ levels (µg/m³) by treatment group and the corresponding *p*-values from the *t*-tests. For the school year (September 2022 through May 2023), the average PM_2.5_ level in treatment classrooms was 39.9% lower than controls classrooms (*p* < 0.001). Compared to baseline values in July 2022 (prior to the start of the intervention), PM_2.5_ levels were reduced on average by 28.7% for HEPA treatment classrooms, compared to only 15.7% for non-HEPA control classrooms. The difference between average PM_2.5_ concentrations in treatment and control groups in July 2022 was not statistically significant (*p* = 0.27). Months with extended student breaks, when students were not present and some air cleaners were turned off, also did not show statistically significant differences: January 2023 (*p* = 0.14), March 2023 (*p* = 0.41), and May 2023 (*p* = 0.12). Figure 1 shows temporal trends, with PM_2.5_ levels peaking in October and April. Similar results were observed for PM_1_ and PM_10_ (Table 2).

**Table 1:** Average monthly and annual PM_2.5_ (µg/m³) during the 2022–2023 school year by treatment group.

PM_2.5_ (µg/m³)	HEPA	Non-HEPA	
Month	Mean (SD)	Mean (SD)	*p*-value
July 2022	1.251 (1.230)	1.742 (3.888)	0.273
August 2022	0.912 (0.796)	1.837 (2.670)	0.003
September 2022	0.813 (0.816)	1.746 (2.479)	0.001
October 2022	1.148 (0.932)	2.226 (3.267)	0.005
November 2022	0.794 (0.882)	1.281 (1.047)	0.001
December 2022	1.250 (1.220)	1.739 (1.166)	0.007
January 2023	0.906 (1.538)	1.255 (1.517)	0.142
February 2023	0.592 (0.583)	1.077 (1.652)	0.014
March 2023	0.579 (1.532)	0.907 (3.283)	0.413
April 2023	1.066 (1.003)	1.868 (2.215)	0.004
May 2023	0.752 (1.043)	1.092 (1.609)	0.117
June 2023	0.605 (0.709)	0.918 (0.790)	0.011
Sept 2022 – May 2023	0.887 (1.116)	1.468 (2.208)	<0.001

**Table 2:** Average monthly and annual PM_10_ (µg/m³) and PM_1_ (µg/m³) during the 2022–2023 school year by treatment group.

	PM_10_ (µg/m³)			PM_1_ (µg/m³)		
	HEPA	Non-HEPA		HEPA	Non-HEPA	
Month	Mean (SD)	Mean (SD)	*p*-value	Mean (SD)	Mean (SD)	*p*-value
Jul 2022	1.297 (1.273)	1.807 (4.238)	0.293	1.205 (1.206)	1.710 (3.875)	0.256
Aug 2022	0.978 (0.836)	1.940 (2.904)	0.004	0.833 (0.755)	1.737 (2.611)	0.003
Sep 2022	0.903 (0.873)	1.866 (2.699)	0.002	0.700 (0.763)	1.611 (2.402)	0.001
Oct 2022	1.250 (0.982)	2.357 (3.537)	0.007	1.026 (0.869)	2.089 (3.222)	0.004
Nov 2022	0.866 (0.914)	1.360 (1.064)	0.001	0.712 (0.847)	1.184 (1.028)	0.001
Dec 2022	1.315 (1.246)	1.805 (1.175)	0.008	1.175 (1.193)	1.652 (1.139)	0.008
Jan 2023	1.003 (1.633)	1.361 (1.651)	0.161	0.802 (1.509)	1.133 (1.442)	0.149
Feb 2023	0.683 (0.645)	1.210 (1.790)	0.014	0.481 (0.520)	0.932 (1.606)	0.018
Mar 2023	0.758 (2.353)	1.000 (3.563)	0.604	0.428 (0.927)	0.823 (3.265)	0.294
Apr 2023	1.206 (1.236)	1.984 (2.383)	0.010	0.921 (0.871)	1.726 (2.168)	0.003
May 2023	0.855 (1.207)	1.175 (1.640)	0.165	0.617 (0.845)	0.990 (1.613)	0.071
Jun 2023	0.693 (0.793)	1.009 (0.830)	0.017	0.518 (0.660)	0.824 (0.772)	0.009
Sep 2022 – May 2023	0.992 (1.323)	1.572 (2.372)	< 0.001	0.771 (0.987)	1.352 (2.170)	<0.001

### Outdoor air data

**Fig. 2:**
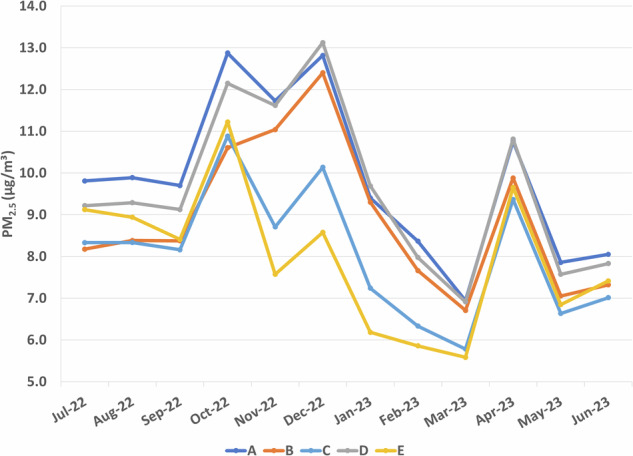
Temporal trends for outdoor PM_2.5_. Monthly outdoor PM_2.5_ (µg/m³) levels over the 2022–23 school year.

Five elementary schools had data available from outdoor Clarity air monitors. Table 3 presents the monthly outdoor PM_2.5_ levels along with the indoor treatment and control classrooms PM_2.5_ levels in those schools. The annual (September to May) outdoor PM_2.5_ levels were higher than the classroom levels. The infiltration of outdoor PM_2.5_ into classrooms was consistently lower in the HEPA classrooms compared to the non-HEPA classrooms, although the confidence intervals overlapped for schools A, B, and D. The difference between infiltration for treatment and control classrooms varied by school, with HEPA classrooms ranging from 13.8% (school B) to 82.4% (school C) lower than non-HEPA classrooms. Ambient PM_2.5_ levels peaked in October and April, similar to indoor levels (Fig. 2). December was also relatively higher, but this was not captured in the indoor data, perhaps due to air monitors being turned off for the winter break.

**Table 3:** Comparison of classroom and outdoor PM_2.5_ (µg/m³) annual averages and infiltration rates by treatment group.

	PM_2.5_ (µg/m³)			Infiltration	
	HEPA	Non-HEPA	Outdoor	HEPA	Non-HEPA
School	Mean (SD)	Mean (SD)	Mean (SD)	Average Ratio (95% CI)	Average Ratio (95% CI)
A	1.921 (2.344)	2.212 (1.272)	10.061 (4.971)	0.171 (0.129, 0.212)	0.217 (0.181, 0.252)
B	0.461 (0.359)	0.540 (0.414)	9.237 (4.190)	0.050 (0.029, 0.070)	0.058 (0.046, 0.071)
C	0.351 (0.370)	2.237 (2.214)	8.152 (3.743)	0.043 (0.035, 0.051)	0.244 (0.167, 0.321)
D	0.810 (0.702)	1.081 (0.845)	9.901 (4.802)	0.081 (0.056, 0.106)	0.127 (0.105, 0.149)
E	0.538 (0.409)	0.934 (0.671)	7.780 (3.724)	0.069 (0.048, 0.091)	0.130 (0.110, 0.151)

## Discussion

Few studies have examined HEPA filtration in a classroom environment. This study aimed to assess if air cleaners using HEPA filters reduced classroom indoor air pollution exposure using a block randomized crossover trial in elementary schools in Los Angeles County that were already using existing HVAC systems with MERV 13 filtration due to the COVID-19 pandemic. In July 2022 before the placement of portable air cleaners, there was no statistically significant difference between PM concentrations in the treatment and control classrooms, indicating successful randomization with regard to baseline PM concentrations. Our findings show that HEPA classrooms had 39.9% lower average annual PM_2.5_ than non-HEPA classrooms (0.887 µg/m^3^ compared to 1.468 µg/m^3^) during the 2022–2023 school year. Outdoor PM_2.5_ infiltration was lower in the HEPA classrooms compared to the non-HEPA classrooms.

A previous study that used a similar study design was conducted from April to December 2021, Mengzhou city, Henan Province, China [15]. PM_2.5_ concentrations were measured in classrooms, living rooms, and outdoor environments during the study period. MicroPEM samplers were also used in the students’ living room. The living room and classroom interventions contributed to 42.31% and 21.34% reductions in personal PM_2.5_ exposure, respectively. Participants with living room and classroom air purification interventions had the lowest PM_2.5_ levels, with an average of 45.9 ± 44.4 µg/m^3^, followed by participants with only living room intervention (62.0 ± 51.5 µg/m^3^), participants with only classroom intervention (73.4 ± 54.1 µg/m^3^), and participants with no intervention (89.0 ± 61.4 µg/m^3^). PM_2.5_ levels in this study were much higher than levels in Los Angeles.

Another air filtration intervention study conducted from 2015 to 2020 in urban elementary schools located in the Northeastern United States utilized a factorial randomized trial with a four-arm design [16]. This study examined treatment with and without air cleaners with HEPA filters and with and without school-wide integrated pest management (IPM). The median PM_2.5_ classroom exposure at baseline was 5.5 µg/m^3^ with HEPA filtration and no IPM and 6.1 µg/m^3^ with control filtration. After the intervention, the median PM_2.5_ in classroom exposure was 3.1 µg/m^3^ and 5.3 µg/m^3^ for HEPA and control filtration, respectively. These PM levels are closer to those measured in the current study and the control classrooms were 70% higher than HEPA treatment classrooms, similar to what we observed (65%).

A third study also studied urban elementary schools from the Northeastern United States from 2013 to 2014, using a pilot randomized controlled trial [17]. Treatment classrooms received HEPA filtration while control classrooms had the filters replaced by a sound device to mimic the noise from the air filtration. Prior to randomization, baseline mean classroom levels of PM_2.5_ were 6.3 μg/m^3^ with no statistically significant differences between the control and treatment classrooms. In the control group, mean PM_2.5_ concentrations decreased from 6.4 μg/m^3^ at baseline to 4.8 μg/m^3^ and 5.0 μg/m^3^ at the first and second follow-up visits, respectively. In the treatment group, mean PM_2.5_ concentrations decreased from 6.2 μg/m^3^ at baseline to 2.4 μg/m^3^ and 2.6 μg/m^3^ at the first and second follow-up visits, respectively. The intervention group had greater reductions in PM_2.5_ levels compared to the control group, corresponding to a 49% and 42% reduction, respectively. This is similar to the reduction of 29.7% observed in the current study, even though the classroom PM_2.5_ levels we observed were much lower. Like the previous results, portable HEPA filter air cleaners were effective in improving short-term air quality in classroom environments. It is unclear whether the classrooms in any of the three previous air cleaner studies had pre-existing HVAC filtration.

Compliance limits the effectiveness of the air cleaners. Since our study used intention-to-treat analysis, its treatment effect estimates are more conservative (i.e., likely to be underestimated if there is imperfect compliance with treatment assignments, such as teachers installing their own HEPA filters in non-HEPA air purifiers). Although our study team noted that the majority of the air cleaners remained powered on with the correct assigned filters in place at the end of the first 6 months, when the filters were replaced, we could not monitor and ensure their proper use throughout the entire year, making ITT analysis the most appropriate choice. However, heterogeneity of results is more likely from mixing non-compliant and compliant data into the final analysis. Another limitation is that the outdoor monitors were installed prior to this study so we do not have calibration data associated with these monitors. The indoor monitors were collocated, and agreement was confirmed prior to placing them in the classrooms.

Interpretation of our results should be made with some consideration. During the study period, the ambient air quality was generally good, ranging from 7.8–10.1 µg/m³ with no wildfire smoke events, so we cannot assess the performance of the additional air filtration during extreme pollution episodes. Furthermore, indoor levels were very low due to the existing HVAC systems with MERV 13 filters in operation. While there are statistically significant reductions in PM levels with the portable HEPA air cleaners, we cannot say if these small reductions have any meaningful health benefits to the students or teachers.

In summary, our results are consistent with previous studies showing that, when properly maintained, portable air cleaners with HEPA filters are effective at removing PM_2.5_ from classrooms [15–17]. The schools in our study were using MERV 13 filters in their HVAC systems during the entire length of our intervention study due to the COVID-19 pandemic, which prevented a considerable about of infiltration of outdoor PM_2.5_ into the classrooms. However, infiltration was still lower in the treatment classrooms with HEPA filters compared to the control classrooms. Our findings provide support for the use of portable air cleaners with HEPA filters in classrooms to reduce PM, even in classrooms with existing HVAC air filtration.
